# Intra-articular tranexamic acid as an adjunct to intravenous tranexamic acid for simultaneous bilateral total knee arthroplasty: a randomized double-blind, placebo-controlled trial

**DOI:** 10.1186/s12891-019-2890-8

**Published:** 2019-10-22

**Authors:** Sachiyuki Tsukada, Kenji Kurosaka, Masahiro Nishino, Tetsuyuki Maeda, Yoshiharu Yonekawa, Naoyuki Hirasawa

**Affiliations:** 1 Department of Orthopaedic Surgery, Hokusuikai Kinen Hospital, 3-2-1 Higashihara, Mito, Ibaraki 310-0035 Japan; 2 Department of Anesthesiology, Hokusuikai Kinen Hospital, Mito, Japan; 3 Department of Nursing, Hokusuikai Kinen Hospital, Mito, Japan

**Keywords:** Knee, Primary arthroplasty, Blood loss, Transfusion, Complication

## Abstract

**Background:**

Intra-articular tranexamic acid (TXA) as an adjunct to intravenous TXA was reported to decrease perioperative blood loss during unilateral total knee arthroplasty (TKA). However, there have been no randomized controlled trials comparing intravenous versus combined intravenous and intra-articular TXA administration in patients undergoing simultaneous bilateral TKA.

**Methods:**

We randomly assigned 77 patients with 154 involved knees undergoing simultaneous bilateral TKA to the intravenous TXA group (intra-articular placebo for each knee) or combined TXA group (1000 mg of intra-articular TXA for each knee) with 1:1 treatment allocation. In both groups, 1000 mg of TXA was given intravenously twice, just before surgery and 6 h after the initial administration. Other perioperative medications, surgical procedures, and blood management strategies were the same for all patients. The primary outcome was perioperative blood loss calculated from blood volume and change in hemoglobin from preoperative to postoperative day 3.

**Results:**

Intention-to-treat analysis showed no statistically significant differences in perioperative blood loss until postoperative day 3 (1067 ± 403 mL in the intravenous TXA group vs. 997 ± 345 mL in the combined TXA group [95% CI, − 240 to 100 mL], *P* = 0.42). No patients required allogenic blood transfusion. The incidence of thrombotic events did not differ between groups (12% in the intravenous TXA group vs. 9% in the combined TXA group; *P* = 0.73).

**Conclusions:**

The addition of intra-articular TXA did not reduce perioperative blood loss in patients undergoing simultaneous bilateral TKA compared with placebo.

**Trial registration:**

University Hospital Medical Information Network UMIN000026137. Registered 14 February 2017.

## Introduction

Although tranexamic acid (TXA) has become an essential component of modern blood preservation strategies for patients undergoing total knee arthroplasty (TKA), the most effective administration route remains arguable [1, 2]. The intravenous route has been the most common route for TXA administration [3]. Meanwhile, intra-articular TXA administration has been shown to have similar effectiveness to intravenous TXA administration regarding blood preservation after TKA [1, 4]. Many recent randomized controlled trials (RCTs) concluded that intra-articular TXA as an adjunct to intravenous TXA could reduce blood loss after unilateral TKA without increasing thrombotic events compared with intravenous TXA administration alone [5, 6]. However, several RCTs reported that perioperative blood loss did not differ between combined intravenous and intra-articular TXA and intravenous TXA alone [7, 8].

The management of perioperative blood loss of simultaneous bilateral TKA is more challenging than unilateral or staged bilateral TKA [9]. Only one retrospective study compared the effectiveness of intravenous TXA and combined intravenous and intra-articular TXA in simultaneous bilateral TKA [10]. Stricter evidence is required to determine the effective administration route of TXA for simultaneous bilateral TKA.

We conducted a double-blind, placebo-controlled RCT to compare the clinical effectiveness of combined intravenous and intra-articular TXA with intravenous TXA alone in patients undergoing simultaneous bilateral TKA. The hypothesis for this RCT was that perioperative blood loss would be lower in patients receiving combined intravenous and intra-articular TXA than in those receiving intravenous TXA alone without increasing thrombotic events.

## Materials and methods

### Trial design

We conducted a single-center, double-blind, clinical superiority RCT in which patients who underwent simultaneous bilateral TKA were randomly assigned in a 1:1 ratio to receive either a combination of 1000 mg of TXA intravenously and 1000 mg of intra-articular TXA in each knee (combined TXA group) or 1000 mg of TXA intravenously and intra-articular normal saline in each knee (intravenous TXA group). The institutional review board provided ethical oversight and study approval. All patients provided written informed consent before inclusion. The study was registered with the University Hospital Medical Information Network, number UMIN000026137.

### Study participants

We recruited participants via one treating clinician (ST) between February 2017 and January 2019. Patients were considered for inclusion if they were 20 years of age or older and medically fit for simultaneous bilateral TKA. The protocol-specified exclusion criteria were patients with known allergic reaction to TXA, patients with preoperative hemoglobin level under 11.0 g/dL, patients who refused blood products, and patients who were enrolled in another interventional clinical trial within 6 months prior to surgery.

To maintain generalizability, we included patients receiving chronic antithrombotic therapy. In such patients, we applied a continued strategy of chronic antithrombotic therapy; chronic antithrombotic therapy with antithrombotic agents, including antiplatelet agents, vitamin K antagonists, and direct oral anticoagulants were continued during the perioperative period, including the day of surgery [11]. Our protocol-specified criteria did not exclude patients with a history of prior thromboembolic events.

### Randomization and blinding

The individual patients were randomly allocated to treatment on a 1:1 basis. We generated a sequence of random numbers from 0 to 99 using computer software (R; The R Foundation for Statistical Computing, Vienna, Austria). We prepared sufficient number of opaque envelopes into which these randomized numbers were placed. A sealed envelope was selected just after starting TKA of the first knee by the allocating staff who were not otherwise involved in the trial. Patients with even numbers were allocated to the intravenous TXA group and those with odd numbers were allocated to the combined TXA group.

### Interventions

The study intervention was intra-articular TXA or placebo as an adjunct to intravenous TXA. Thus, the study treatments were combined intravenous and intra-articular TXA or intravenous TXA and intra-articular normal saline. The study drugs were identical in appearance because the TXA solution was transparent. All other elements of perioperative interventions remained the same for all both groups.

In the combined TXA group, patients received 1000 mg of TXA intravenously (Transamin; Daiichi-Sankyo, Tokyo, Japan) just before the skin incision in the first knee. After implantation of the prosthesis, we closed the capsule and retinaculum. Then, we injected 1000 mg of TXA (10 mL of 100 mg/mL TXA) intra-articularly into each knee through the medial patellar retinaculum using a 23-gauge needle [10]. Thus, a total of 3000 mg of TXA was administered in the operating theater for patients allocated to the combined TXA group. In the ward, another 1000 mg of TXA was given intravenously 6 h after the initial intravenous administration.

In the intravenous TXA group, 1000 mg of TXA was similarly administered intravenously just before the skin incision in the first knee. After closing the capsule and retinaculum, 10 mL of normal saline was administered intra-articularly into each knee. For the patients allocated to the intravenous TXA group, 1000 mg of TXA was administered in the operating theater. Six hours later, we administered another 1000 mg of TXA intravenously.

### Pre- and postoperative medication

No thromboprophylaxis was prescribed to prevent venous thromboembolism during the study period. For patients who had chronic antithrombotic therapy with antithrombotic agents, all antithrombotic agents were continued during the perioperative period [11].

Antibiotic prophylaxis with 1000 mg of cefazolin (Cefamezin; Astellas, Tokyo, Japan) was intravenously administered perioperatively.

All patients received an intraoperative periarticular injection of an analgesic solution containing 40 mL of ropivacaine (Anapeine, 7.5 mg/mL; AstraZeneca, Osaka, Japan), 1.0 mL of morphine hydrochloride hydrate (10 mg/mL; Takeda), 0.6 mL of epinephrine (Bosmin, 1.0 mg/mL; Daiichi-Sankyo), 80 mg of methylprednisolone (Sol Mercort; Fuji, Toyama, Japan), and 50 mg of ketoprofen (Capisten; Kissei, Nagano, Japan) [12]. These agents were mixed with normal saline solution to achieve a combined volume of 120 mL, and 60 mL of the mixture was injected into each knee [12]. For each knee, 40 mL of solution was injected into the extensor mechanism, pes anserinus, and anteromedial capsule just prior to arthrotomy, and the remaining 20 mL of solution was injected into the posterior capsule, posteromedial structures, and periarticular synovium just before implantation [13].

All patients received 4 mg of oral non-steroidal anti-inflammatory drug (lornoxicam, Lorcam; Taisho-Toyama, Tokyo, Japan) three times a day for postoperative pain relief.

### Anesthesia, surgery, and rehabilitation

All simultaneous bilateral TKAs were performed sequentially with one surgical team under the same session of anesthesia; the operation on the second knee started after completion of wound closure on the first side.

All patients were managed with general anesthesia induced using short-acting volatile anesthetic (sevoflurane; Sevofrane, Maruishi, Tokyo, Japan) and intravenous anesthetic (propofol, Diprivan; AstraZeneca, Osaka, Japan). Anesthesia was maintained with sevoflurane and continuous infusion of short-acting opioid (remifentanil, Ultiva; Janssen, Tokyo, Japan). The details of intraoperative blood pressure management were left to the discretion of the anesthesiologists. The study protocol prohibited regional anesthesia.

All surgical procedures were performed by one of two orthopedic surgeons (ST and KK). No pneumatic tourniquet was used during the study period. An anterolateral incision was used in all surgeries [14]. A subvastus approach without patellar eversion was used except in patients with valgus knee alignment, for whom a lateral approach was used. All patients received a cemented, posterior-stabilized prosthesis. Intraoperative bone cutting and soft tissue balancing were performed using standardized techniques as reported previously [15]. We used intramedullary guides for femoral preparations and plugged the femoral canal with autologous bone. No drain was placed for any of the patients. An intermittent foot-pump system was routinely used to prevent deep vein thrombosis (DVT) before the patient began walking exercise. All patients underwent the same standardized postoperative rehabilitation regimen from the day after TKA.

### Blood management strategies

The blood management strategies were performed in accordance with standardized methods as reported previously [10]. The study protocol included predonation of 800 mL or 400 mL of autologous blood for patients scheduled for simultaneous bilateral TKA. In patients who predonated 800 mL of blood, we collected 400 mL at 4 weeks before the TKA and another 400 mL at 2 weeks before the TKA. In patients who predonated 400 mL of blood, we collected 400 mL at 2 to 3 weeks before the TKA.

No predonated autologous blood was discarded during the study period. For patients who predonated 800 mL of blood, we routinely returned half (400 mL) of the 800-mL predonation of autologous blood to the patient on the day of the simultaneous bilateral TKA and returned the remaining 400 mL on the day after TKA. For patients who predonated 400 mL of blood, we returned all 400 mL on the day of the TKA.

We did not employ any intraoperative blood salvage technique.

We planned additional allogenic blood transfusion for patients with a hemoglobin level of < 7.0 g/dL who were asymptomatic and those with a hemoglobin level of < 10.0 g/dL who had symptoms related to anemia.

### Pre- and postoperative screening for venous thromboembolism

Plasma D-dimer level was measured as routine preoperative laboratory testing. Patients with plasma D-dimer level >  0.5 μg/mL were routinely tested for DVT by skilled clinical laboratory technicians using ultrasonography.

At 1 day after TKA, all patients were screened for the presence of DVT by ultrasonography. At 7 days after TKA, patients were screened using the clinical model of Wells et al. [16], and those with a score of 3 or higher were again tested by ultrasonography. Pulmonary embolism was diagnosed based on clinical symptoms and enhanced chest computed tomography.

### Outcome measures

#### Primary outcome

The prespecified primary outcome was the volume of perioperative blood loss, which was measured using the calculated blood volume and change in hemoglobin from preoperative to postoperative day 3 [10]. The blood volume of each patient was calculated using the formula reported by Nadler et al. [17]. For patients who received predonated autologous blood, we used the hemoglobin level prior to donation for this calculation. For patients who received allogenic blood, we planned to use a hemoglobin level of 19 g/dL and 140 mL for 1 unit according to the data of the Japanese Red Cross Society.

The routine length of hospital stay in our institute was more than 7 days for all patient undergoing simultaneous bilateral TKA. No patients were unnecessarily hospitalized with the aim of measurements.

### Secondary outcomes

The calculated blood loss at 7 days after TKA and the number of patients requiring allogeneic blood transfusion were compared between the two groups. Major bleeding and thrombotic events up to 3 months after TKA were investigated with special reference to DVT. Major bleeding and thrombotic events were defined according the criteria of Mantz et al. [18].

### Sample size calculation

We hypothesized that the combined TXA group showed superior effectiveness to the intravenous TXA group in terms of perioperative blood loss. A previous RCT reported that the postoperative blood loss was lower, with mean value of 410 mL (95% confidence interval, 300 to 525 mL) in the patients receiving combined intravenous and intra-articular TXA than patients receiving no TXA in simultaneous bilateral TKA [19]. The panel reached a consensus that the minimum value of 95% confidence interval of that study (300 mL) was employed for the sample size calculation of our study since there was no prior study clearly identifying what a minimally clinically important difference would be for the reduction of perioperative blood loss after simultaneous bilateral TKA. Thus, we powered this superiority trial to detect a 300-mL difference in perioperative blood loss between the combined TXA group and intravenous TXA group. Based on a type I error rate of 5% and a power of 80%, we calculated that a minimum of 32 patients would be required in each group. For power analysis, we used a standard deviation of 425 mL in perioperative blood loss noted in observational pilot data.

### Missing data and statistical analysis

Prior to starting this RCT, we determined to use a complete case analysis for missing data of primary outcome as the number of missing values was predicted to be small for primary outcome [20].

Differences between treatment groups were assessed on an intention-to-treat basis. Differences in primary outcome between the two treatment groups were analyzed by Student’s t-test. In addition, we calculated 95% confidence intervals by comparing outcomes in the combined TXA group with intravenous TXA group. To evaluate the impact of the continuing chronic antithrombotic therapy, the volume of the perioperative blood loss until postoperative day 3 was also compared between four groups using analysis of variance: the combined TXA group with and without chronic antithrombotic therapy and the intravenous TXA group with and without chronic antithrombotic therapy.

For secondary outcomes and baseline characteristics, data were compared between the groups with Student’s t-test for continuous variables, which are presented as means and standard deviations, and Fisher’s exact test for categorical variables, which are presented as numbers and percentages.

A 2-sided *P* value of 0.05 was considered to indicate statistical significance. All analyses were conducted using R software.

## Results

**Fig. 1:**
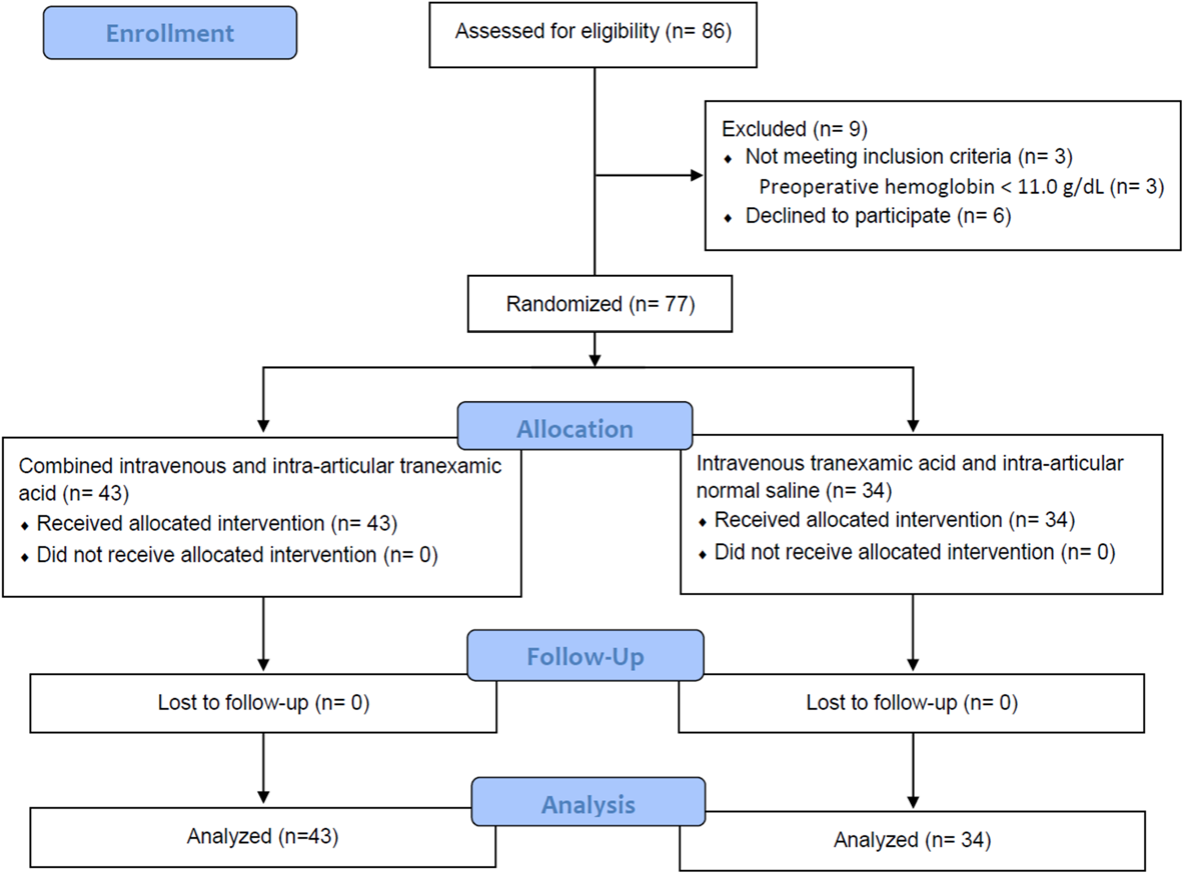
Consent, randomization, and follow-up of participants in the randomized controlled trial comparing intravenous versus combined intravenous and intra-articular tranexamic acid for simultaneous bilateral total knee arthroplasty

Figure 1 shows the flow of these patients through the trial. A total of 77 patients consented to take part in the trial and underwent randomization: 43 to the combined TXA group and 34 to the intravenous TXA group. All analyses were conducted before the randomization code was broken. We obtained all data for the primary outcome of this study without missing data.

Table 1 summarizes the demographic characteristics of patients at baseline. Although we did not set the exclusion criteria for preoperative diagnosis, preoperative diagnoses were osteoarthritis of the knee for all patients included in this study. Mean volume of intraoperative blood loss was 117 ± 68 mL for one knee (*n* = 154). There was no difference between two surgeons in terms of intraoperative blood loss (121 ± 73 m L vs. 101 ± 40 mL [95% confidence interval of the difference, − 58 to 18 mL], *P* = 0.30).

**Table 1:** Patient demographic and baseline clinical characteristics

	Combined intravenous and intra-articular tranexamic acid (*n* = 43)	Intravenous tranexamic acid and intra-articular normal saline (*n* = 34)	*P-*value
Age, years	75 ± 6	77 ± 6	0.22*
Sex (female/male)	33/10	28/6	0.57†
Body mass index, kg/m^2^	27.7 ± 3.8	28.1 ± 4.2	0.66*
Receiving chronic antithrombotic therapy (yes/no)	8/35	5/29	0.76†
History of Diabetes Mellitus (yes/no)	9/34	6/28	0.78†
Preoperative hemoglobin, g/dL	13.2 ± 1.3	12.9 ± 1.1	0.34*
Deep venous thrombosis detected with preoperative screening (yes/no)	1/42	0/34	> 0.99*

Results are expressed as means ± standard deviation, unless otherwise stated

*P-values were determined with Student’s *t* test

†*P*-values were determined with the Fisher’s exact test

### Primary outcome

Intention-to-treat analysis showed that the volume of perioperative blood loss did not differ between treatment groups at 3 days after TKA (997 ± 345 mL in the combined TXA group vs. 1067 ± 403 mL in the intravenous TXA group [95% confidence interval of the difference, − 240 to 100 mL], *P* = 0.42). We found no statistically significant differences between patients with and without chronic antithrombotic therapy (996 ± 360 mL in the combined TXA group without chronic antithrombotic therapy vs. 1005 ± 292 mL in the combined TXA group with chronic antithrombotic therapy vs. 1006 ± 406 mL in the intravenous TXA group without chronic antithrombotic therapy vs. 1419 ± 91 mL in the intravenous TXA group with chronic antithrombotic therapy, *P* = 0.11).

### Secondary outcomes

We saw no evidence of treatment group effects for any secondary outcome measures. The volume of perioperative blood loss until postoperative day 7 was not different between the two groups (1147 ± 446 mL in the combined TXA group vs. 1144 ± 346 mL in the intravenous TXA group [95% confidence interval of the difference, − 182 to 189 mL], *P* = 0.97). No patients required allogenic blood transfusion. There were no major bleeding events in study patients. Distal DVT was detected in four of 43 patients (9%) in the combined TXA group and four of 34 patients (12%) in the intravenous TXA group (*P* = 0.73). None of the study patients experienced major thrombotic events other than distal DVT.

## Discussion

This double-blind, placebo-controlled RCT showed that additional intra-articular TXA to intravenous TXA was no more effective than placebo in reducing perioperative blood loss in patients undergoing simultaneous bilateral TKA.

This is the first RCT to compare the effectiveness of combined intravenous and intra-articular TXA and that of intravenous TXA alone for patients undergoing simultaneous bilateral TKA. Although TXA has been widely used in TKA to reduce perioperative blood loss [1, 21], the most effective route of administration for TXA remains moot [2]. In unilateral TKA, there has been conflicting evidence regarding the benefit of adding intra-articular TXA to intravenous TXA for perioperative blood preservation [5–8]. The results of our study did not support the use of intra-articular TXA as an adjunct to intravenous TXA.

The key limitation of this study was that this was a single-center study. There were specific features of our clinical practice, such as surgery without use of tourniquet and inpatient procedure. However, several investigators underscored that the effectiveness of TXA should be studied in the setting of TKA without use of a tourniquet as the modern fast-track setup must include both no use of tourniquet and effective administration of TXA [5, 10]. Moreover, the longer length of hospital stays compared with North America and Europe empowered us to perform routine blood tests at 3 days after TKA and DVT screening at 1 and 7 days after TKA for all study patients. It should be noted that this study excluded patients in whom the preoperative hemoglobin level was under 11.0 g/dL as study protocol included the predonation of the autologous blood. Our findings, especially in terms of the incidence of patients requiring allogenic transfusion, would not be applicable to these patients. The sample size of this RCT allowed the assessment of total blood loss between groups. However, this RCT had limited power for the incidence of thrombotic events because the incidence was low.

The main strength of this study was the use of a randomized placebo-controlled design. One before-and-after study reported that combined intra-articular and intravenous TXA significantly reduced the perioperative blood loss compared to patients treated only with intravenous TXA in simultaneous bilateral TKA [10]. We believe that the randomized placebo-controlled design may bring different results from the before-and-after study as other previous RCTs revealed unexpected results [22–24]. We maintained high adherence to the study protocol and successful blinding of the patients, surgical teams, and outcome assessors and successful randomization and patient allocation concealment. We did not exclude patients with a history of prior thromboembolic events in this RCT. Recently published joint guidelines by five medical associations raised problems about the lack of high-level evidence to support the use of TXA in patients with a history of thromboembolic events [21].

## Conclusion

This RCT showed that intra-articular TXA as an adjunct to intravenous TXA for simultaneous bilateral TKA did not result in decreased postoperative blood loss compared with placebo contrary to the study hypothesis.

## Data Availability

The datasets used and/or analysed during the current study are not publicly available. Data are however available from the corresponding author on reasonable request.

## Abbreviations

DVT: Deep vein thrombosis
RCT: Randomized controlled trial
TKA: Total knee arthroplasty
TXA: Tranexamic acid
